# THz pulse doubler at FLASH: double pulses for pump–probe experiments at X-ray FELs

**DOI:** 10.1107/S1600577517015442

**Published:** 2018-01-01

**Authors:** Ekaterina Zapolnova, Torsten Golz, Rui Pan, Karsten Klose, Siegfried Schreiber, Nikola Stojanovic

**Affiliations:** a Deutsches Elektronen-Synchrotron – DESY, Notkestrasse 85, D-22607 Hamburg, Germany

**Keywords:** FLASH, free-electron laser, THz beamline, pump–probe experiments, double electron bunches, X-rays

## Abstract

FLASH, the X-ray free-electron laser in Hamburg, Germany, employs a narrowband high-field accelerator THz source for unique THz pump X-ray probe experiments. However, the large difference in optical paths of the THz and X-ray beamlines prevents utilization of the machine’s full potential (*e.g.* extreme pulse energies in the soft X-ray range). To solve this issue, lasing of double electron bunches, separated by 28 periods of the driving radiofrequency (at 1.3 GHz), timed for the temporal overlap of THz and X-ray pulses at the experimental station has been employed. In order to optimize conditions for a typical THz pump X-ray probe experiment, X-ray lasing of the first bunch to one-sixth of that of the second has been suppressed. Finally, synchronization of THz radiation pulses was measured to be ∼20 fs (r.m.s.), and a solution for monitoring the arrival time for achieving higher temporal resolution is presented.

## Abstract 2

Double electron bunch lasing at an X-ray free-electron laser for temporal overlap in pump–probe experiments is presented.

## 1. Introduction   

The X-ray free-electron laser FLASH1 at DESY in Hamburg, Germany, has a worldwide unique feature – a THz undulator behind the main X-ray undulator section, that allows users to perform pump–probe experiments with ultrashort high-intensity THz and soft X-ray pulses (Gensch *et al.*, 2008 ▸; Frühling *et al.*, 2009 ▸; Tavella *et al.*, 2011 ▸; Stojanovic & Drescher, 2013 ▸; Oelze *et al.*, 2017 ▸). The wavelength range can be tuned from 1 to 300 µm (1 to 300 THz), depending on the electron beam energy. THz and X-ray pulses are produced by the same electron bunch, and are naturally synchronized (down to 5 fs) (Frühling *et al.*, 2009 ▸; Tavella *et al.*, 2011 ▸). However, a problem that plagues this type of experiment is that THz and X-ray optical paths are significantly different. In the beamline, the THz beam is collimated using five toroidal mirrors to keep the beam size within the size of the beam transport and optics (size 210 mm × 150 mm). Due to the extremely broad spectral range that the undulator covers, all optical elements are reflective (providing spectrally non-dispersive reflection) and provide a 45° angle of incidence. Folding of the THz beam adds additional optical path, adding up to a delay of 21.5 ns (∼6.5 m of optical path) with respect to X-ray pulses at the experimental station. One solution to ensure temporal overlap between THz and X-ray pulses at the experiment is to introduce additional optical path for the X-rays in back-reflection geometry (Feldhaus *et al.*, 2005 ▸; Frühling *et al.*, 2009 ▸; Oelze *et al.*, 2017 ▸) using normal-incidence multilayer mirrors specially designed for a particular wavelength (Bajt *et al.*, 2007 ▸). Although this method has been successfully demonstrated (Frühling *et al.*, 2009 ▸; Oelze *et al.*, 2017 ▸), there are two major drawbacks when utilizing this technique (within the specificities of the discussed setup): low reflectivity and narrow spectral range of the X-ray mirrors. Although not a limitation of the multilayer mirror design, this geometry also suffers from a large focal size of the X-ray beam (>100 µm FWHM), caused by the large focal length (3.5 m) determined by the difference in optical path between THz and X-ray pulses. Reduction of the X-ray fluence in the experiment due to the combined effect of low reflectivity and large focus excludes high-intensity experiments [*e.g.* plasma physics (Nagler *et al.*, 2009 ▸) or non-linear multiphoton ionizations (Richter *et al.*, 2009 ▸, 2010 ▸)] thus limiting FLASH’s high pulse energy advantage (Rönsch-Schulenburg *et al.*, 2017 ▸) over other sources in the same spectral range. An alternative approach which avoids the need of introducing a delay for the X-ray pulses, proposed by Grimm *et al.* (2006 ▸), is to exploit the radiation of tailored electron bunches. In particular, we generate double electron bunches at the electron gun, separated by 21.5 ns. In this scheme the first electron bunch is used to generate THz and the second to generate soft X-ray pulses. Each double electron bunch is generated by two consecutive laser pulses illuminating the photocathode at FLASH’s electron gun.

**Figure 1:**
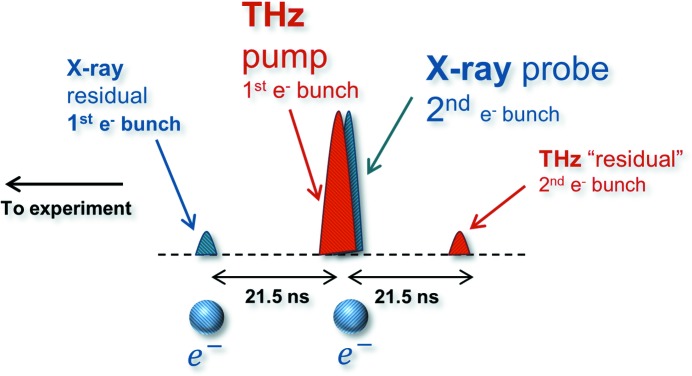
THz doubler. Schematic representation of the X-ray and THz pulse arrival times for the double electron bunch scheme.

For typical THz pump/X-ray probe experiments the first electron bunch needs to be optimized to generate maximal THz pulse energy (refer to scheme in Fig. 1 ▸), while generating little or no X-rays, to reduce their potential influence on the sample. The second bunch, on the other hand, should be optimized to generate X-ray pulses with required properties, ranging from short pulse duration to high pulse energy, while suppressing the THz output (although this is less critical).

In this work we present the generation of THz pulses from double electron bunches, studying the energy-ratio control of individual THz and X-ray pulses and, most importantly, the arrival-time stability of two THz doubler pulses essential for accurate pump–probe experiments. By doing this we are opening the pathway to utilizing the full potential of FLASH’s high pulse energies in the synchronized THz/X-ray pump–probe experiments.

## 2. Concept of double-bunch generation   

**Figure 2:**
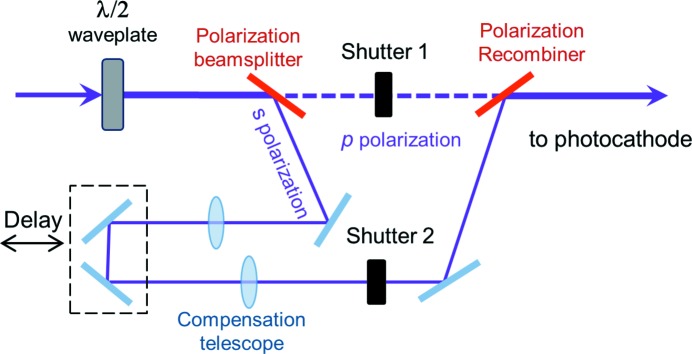
Scheme of the laser pulse doubler [adapted from Grimm *et al.* (2006 ▸)]. The laser pulse is split, one pulse is delayed and then recombined with a direct one. Each branch includes a shutter, so that each pulse can be selected at will.

The concept of the double pulse generation at FLASH (THz doubler) has been demonstrated elsewhere (Grimm *et al.*, 2006 ▸) and the main issues for transport through the accelerator have been identified. Here, we briefly refer to it for completeness (refer to Fig. 2 ▸).

The fourth harmonic of the driving photocathode laser, at 262 nm, is split by a polarization beamsplitter (fused silica at the Brewster angle). One of the branches is delayed by 21.5 ns with the option to fine-tune the arrival time of the second pulse *via* a delay stage (*e.g.* for adjusting it to the phases of the accelerating modules). Divergence and size resulting from the large difference in optical path are compensated for by a Keplerian telescope. The two beams are recombined by a polarization recombiner and brought together at a photocathode. Each of the pulses can be blocked by closing the corresponding shutter.

## 3. Characterization of THz doubler radiation   

Double electron bunches for the THz doubler have been set up following the procedure described by Grimm *et al.* (2006 ▸). Electron bunch charges were almost equal and their arrival time with respect to the accelerating radiofrequency (RF) was adjusted to facilitate electron beam transmission through the accelerator.

In the following we present the characterization of THz and X-ray pulse energies and a novel technique to determine the relative arrival time of individual THz pulses. The X-ray pulse arrival time for the same electron bunch is locked to the respective THz pulse down to a few femtoseconds due to natural synchronization (Frühling *et al.*, 2009 ▸; Tavella *et al.*, 2011 ▸; Oelze *et al.*, 2017 ▸).

### 3.1. THz pulse energy measurements   

The THz undulator at FLASH was set to 140 µm wavelength and the individual electron bunches had charges of 0.3 nC (direct, first) and 0.25 nC (delayed, second). We measured THz pulse energies using a radiometer [RM3700, head RjP-735/RF, by Laser Probe, cross-referenced to PTB traceable 3A-P-THz radiometer (Green *et al.*, 2016 ▸)]. The detector had a cavity pyroelectric probe and its time constant of 1 ms prevented us from resolving individual pulses of the THz doubler. Thus, pulse energies were measured as an average over a few hundred pulses. Selection of individual THz doubler pulses was achieved by closing the corresponding shutter in the injector setup. The first pulse (shutter 1 open, shutter 2 closed) measured 1.05 ± 0.07 µJ, the second pulse (shutter 1 closed, shutter 2 open) measured 0.84 ± 0.06 µJ, and both pulses together measured 1.94 ± 0.13 µJ. Hereby we confirm that electron bunches with similar charge and experiencing the same accelerator conditions (*e.g.* compression) yield similar THz pulse energies.

In the future, we plan to explore adjusting the relative pulse energies in the THz doubler and the influence on the SASE levels. One possibility is to adjust the ratio of the pulse energies illuminating the photocathode by adjusting the polarization of the laser pulse impinging on the beamsplitter in the laser doubler setup. The second option is by adjusting the arrival time of the second pulse with respect to the RF phase in the accelerating module, utilizing the optical delay stage in the respective branch of the photocathode laser.

### 3.2. SASE pulse energy measurements   

**Figure 3:**
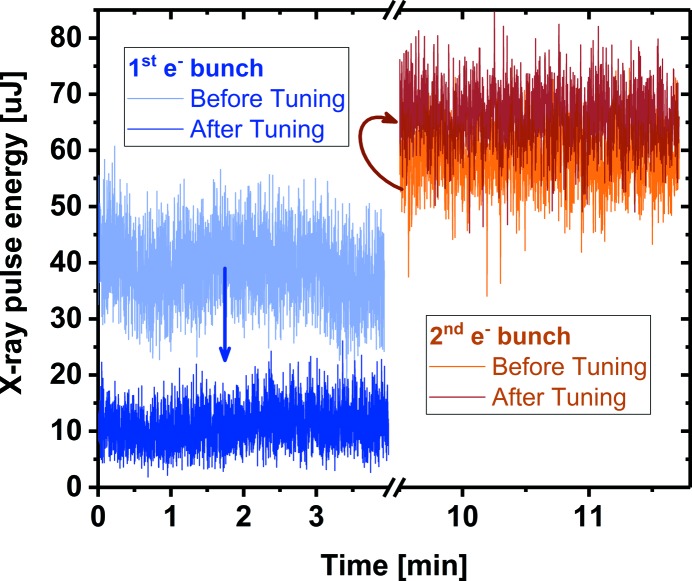
X-ray SASE pulse energies for the first and second pulse of the THz doubler. From initially similar levels, the pulse energy of the first bunch has been suppressed to one-sixth of the intensity of the second.

X-ray pulse energies are measured using one of the gas monitor detectors installed at FLASH1 (Tiedtke *et al.*, 2008 ▸). Temporal resolution of these detectors is in the region of 100 ns. Thus individual X-ray pulses generated by the THz doubler cannot be resolved. The average ratio of the X-ray pulse energy is retrieved by successively measuring the first and second pulse, as for the THz pulses before. The pulse energy of one bunch was measured by blocking the other branch in the electron gun laser. Fig. 3 ▸ displays pulse energy measurements for the first and second pulses. Initially, the first electron bunch, with 0.3 nC charge, produced 40 ± 6 µJ, while the second, with 0.25 nC, produced 58 ± 5 µJ. After optimization by shifting the relative arrival time of each individual laser doubler pulse to the RF phase, the average energy of the first and the second pulses was 11 ± 4 µJ and 67 ± 6 µJ, respectively. These results show the maximum achieved suppression of the first SASE resulting in a ratio of 1:6.

There are several ways to further suppress X-ray lasing of the first bunch. For example, by introducing a slight kick (∼100 µm in the vertical direction) in the electron bunch orbit in the X-ray undulators, it has been shown that the SASE process can be suppressed by two orders of magnitude (Först *et al.*, 2011 ▸), while maintaining similar pulse energies in the THz spectral range (refer to §S3 in the supporting information).

### 3.3. Arrival-time stability   

**Figure 4:**
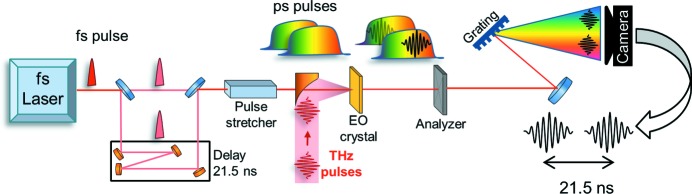
Principle of double THz pulses arrival-time measurement based on the spectral decoding technique.

Electro-optic spectral decoding (EOSD) detection (Jiang & Zhang, 1998 ▸; Steffen, 2007 ▸) maps the THz field onto a temporally chirped probing laser pulse in a single shot. It can be used as an arrival-time marker of THz to the external laser (Kovalev *et al.*, 2017 ▸). We adapt this technique to detect the arrival time of the THz doubler pulses simultaneously (refer to Fig. 4 ▸) using a single probe laser pulse. The laser pulse (30 fs FWHM at 860 nm) is split by a non-polarizing beamsplitter and one pulse is delayed by 21.5 ns. Pulses are then recombined, using the same type of non-polarizing beamsplitter, and follow the same optical path thereafter. Since the laser pulse has been split optically, temporal instabilities are minimal. Both laser pulses are stretched in a 450 mm glass (SF57) stretcher to linearly chirped pulses with 16.5 ps FWHM duration. The stretched pulses are overlapped (spatially and temporally) with THz doubler THz pulses in an electro-optic (EO) crystal.

**Figure 5:**
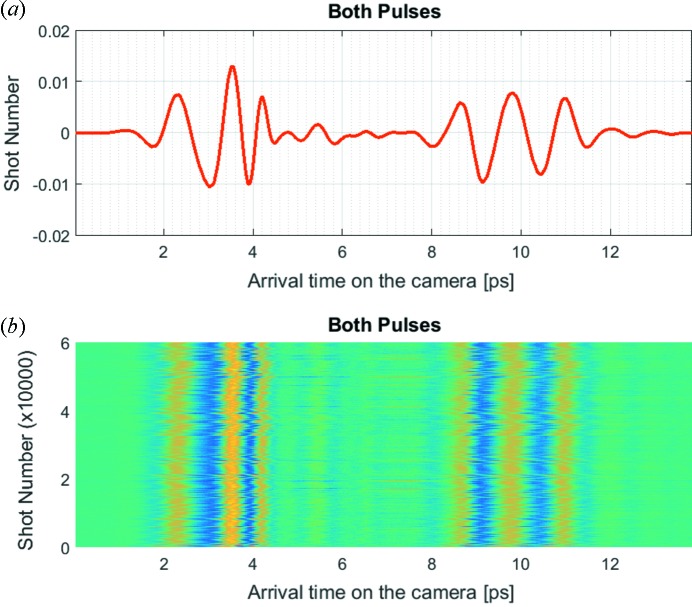
Spectral decoding detection of the THz doubler pulses generated by an electron beam dump magnet (edge radiation). (*a*) Single-shot trace, modulations with the first and the second THz pulse well separated. (*b*) Series of single-shot measurements acquired over ∼90 min (60000 shots).

The electric field of the THz pulses induces birefringence in the EO crystal (1 mm ZnTe) that modulates polarization of the probing laser pulses. Polarization modulation is transferred into a modulation of intensity by an analyser (Wollaston prism). Laser pulses are sent to a grating spectrometer (Princeton Instruments, Acton SP2300i) and detected using a line detector (Basler Sprint Mono spL-2048-70km). The detector response is slower than the time delay between the pulses, which is used to observe the traces of both pulses in the same spectral recording. In order to be able to detect the respective arrival time between the two THz doubler bunches, the arrival time of the probe laser pulses is adjusted so that the THz doubler pulses modulate different spectral regions of their respective probe laser pulses (refer to Fig. 5 ▸ and §S1 of the supporting information). The modulations are well separated to avoid ambiguities in arrival-time determination.

The THz pulses in this example are generated by FLASH’s electron beam dump magnet by the edge radiation process (Geloni *et al.*, 2009 ▸; Tavella *et al.*, 2011 ▸; Stojanovic & Drescher, 2013 ▸). The profile of single-cycle THz pulses measured by EOSD deviates from our previous results [*e.g.* by THz streaking (Stojanovic & Drescher, 2013 ▸) or EO sampling (Golz *et al.*, 2018 ▸)], mainly because of a characteristic of this technique that the frequency mixing between the short THz pulse and the chirped laser pulse leads to a detection distortion (Jamison *et al.*, 2008 ▸; Pan, 2015 ▸). However, the measured distorted profiles keep the same shape within the timing jitter range, and therefore the arrival time of the pulses is not affected due to causality (also confirmed during temporal calibration of the EOSD; refer to §S2 of the supporting information).

**Figure 6:**
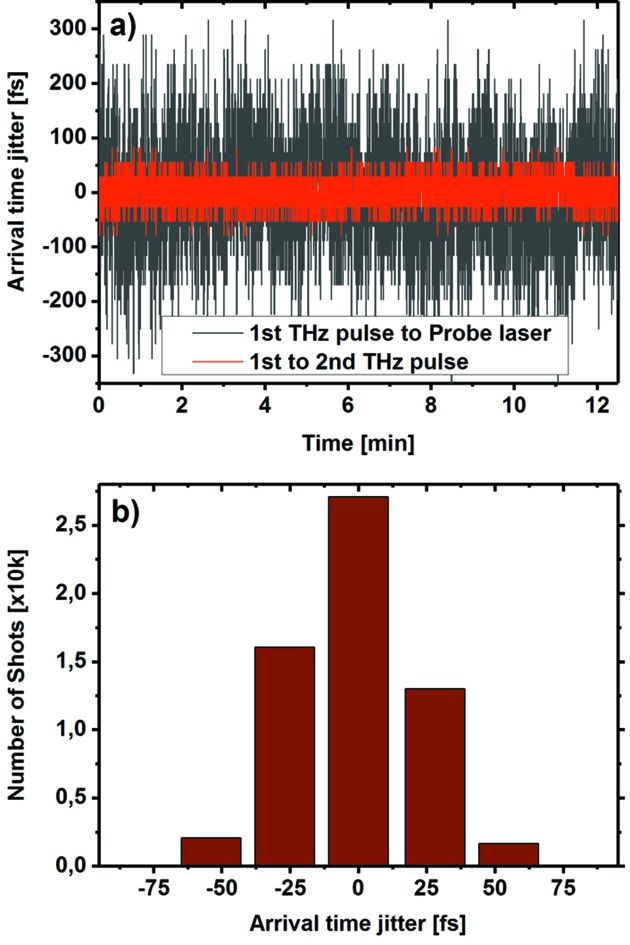
(*a*) Arrival-time jitter of the first THz pulse with respect to the laser (black), and jitter between the two THz pulses (red). (*b*) The distribution of the jitter between individual THz doubler pulses has a width of 23 fs (r.m.s.).

A typical series of subsequent shots is presented in Fig. 6(*a*) ▸. From each trace we extract the arrival time of each individual THz doubler pulse with respect to the corresponding probe laser pulse [black curve in Fig. 6(*a*) ▸]. Their difference gives their relative temporal jitter [red curve in Fig. 6(*a*) ▸].

The overall level of jitter measured over a period of 12 min is estimated to be 23 fs (r.m.s.). We observe that jitter between THz pulses and probing laser (96 fs r.m.s.) is much larger than that between THz doubler pulses. We also performed jitter measurements for the THz undulator set to 140 µm wavelength. The jitter between narrowband THz doubler pulses is estimated to be on the level of 18 fs (r.m.s.) over a period of 15 min.

## 4. Conclusion   

We have demonstrated the feasibility of the THz doubler concept, accelerating and lasing of the double electron bunches timed for the temporal overlap of THz and X-ray pulses in user experiments. In the case of FLASH, electron bunches are separated by 28 periods of the driving RF (at 1.3 GHz) or 21.5 ns. We observed THz pulse energies in the microjoule range and, critically, we have demonstrated suppression of the X-ray pulse energy of the first electron bunch (11 µJ), down to a level of 16% of the second bunch (67 µJ) of the THz doubler. This will significantly widen the range of experiments that can utilize this setup. This suppression can be further improved (by two orders of magnitude) if fast kickers are employed to deflect the electron bunch orbit by 100 µm in the X-ray undulators. Finally, we observed that the arrival-time stability of individual THz pulses in the THz doubler is around 20 fs (r.m.s.). For experiments where a more accurate arrival time is required, spectral decoding presented in this work provides a photon-efficient tool for monitoring the arrival time for data sorting (Azima *et al.*, 2009 ▸; Kovalev *et al.*, 2017 ▸). Since the tool requires only modest laser power (*e.g.* femtosecond oscillator), it can be readily applied at any similar facility (Svetina *et al.*, 2016 ▸) or can be extended for use at larger-scale X-ray free-electron lasers like the European XFEL or LCLS II. The THz doubler has the potential to solve the issue that any of the X-ray free-electron lasers with an integrated THz source will face: a large difference in the optical path between X-rays and accelerator-based THz pulses.

## 5. Related literature   

The following reference, not cited in the main body of the paper, has been cited in the supporting information: Behrens *et al.* (2010) ▸.

## Supplementary Material

Notes on arrival time, temporal calibration and THz pulse energy. DOI: 10.1107/S1600577517015442/xn5009sup1.pdf
