# Supplementary material for: THz pulse doubler at FLASH: double pulses for pump–probe experiments at X-ray FELs
Source: J Synchrotron Radiat. 2018 Jan 1;25(Pt 1):39–43. doi: 10.1107/S1600577517015442 (PMC5741119; doi:10.1107/S1600577517015442)
Supplement: Supplementary file 1 [file s-25-00039-sup1.pdf]

JOURNAL OF SYNCHROTRON RADIATION

Volume 25 (2018)

Supporting information for article:

THz pulse doubler at FLASH: double pulses for pump–probe experiments at X-ray FELs

Ekaterina Zapolnova, Torsten Golz, Rui Pan, Karsten Klose, Siegfried Schreiber and Nikola Stojanovic

S1. Arrival time

Measured traces are separated (i.e. at ~7ps) in the spectrum and arrival time is evaluated for each THz doubler pulse separately. The first trace in a measured set is taken as a nominal arrival time reference. Each subsequent trace is digitally cross-correlated to the reference. The time lag of maximum correlation marks the arrival time change with respect to the reference. Difference of these two arrival times provides the relative arrival time between THz doubler pulses.

S2. Temporal calibration

Temporal calibration is performed by shifting the delay of the laser pulse by 100 µm steps (333fs) with respect to the THz pulse. Thus THz signal was imprinted on different spectral components at each step. At each step, several hundred single-shot measurements were acquired and arrival time averaged. Step size was larger than jitter (See Fig. 6). Arrival time as function of delay (translation stage position) is linear with high degree of linearity. The calibration constant was found to be 27.4 ± 1 fs per camera pixel. THz pulse energy during optimization of 1st SASE pulse

S3. THz pulse energy during suppression of SASE lasing for 1st electron bunch

While direct THz pulse energy measurement was not available during optimization of the 1st SASE pulse energy, relative levels were monitored via electron beam compression monitor (that measure coherent synchrotron radiation in THz range), installed after the last electron bunch compressor (Behrens *et. al.* 2010). Readings before and after 1st SASE pulse optimization were 0.35 and 0.27 respectively, which are within pulse-to-pulse fluctuations. This leads us to a conclusion that THz pulse energy, which is mainly influenced by electron bunch compression, was not significantly affected.
